# Host restriction factors in retroviral infection: promises in virus-host interaction

**DOI:** 10.1186/1742-4690-9-112

**Published:** 2012-12-20

**Authors:** Yong-Hui Zheng, Kuan-Teh Jeang, Kenzo Tokunaga

**Affiliations:** 1 Department of Microbiology and Molecular Genetics, Michigan State University, East Lansing, MI, USA; 2 The National Institutes of Health, Bethesda, MD, USA; 3 Department of Pathology, National Institute of Infectious Diseases, Tokyo, Japan

## Abstract

Retroviruses have an intricate life cycle. There is much to be learned from studying retrovirus-host interactions. Among retroviruses, the primate lentiviruses have one of the more complex genome structures with three categories of viral genes: structural, regulatory, and accessory genes. Over time, we have gained increasing understanding of the lentivirus life cycle from studying host factors that support virus replication. Similarly, studies on host restriction factors that inhibit viral replication have also made significant contributions to our knowledge. Here, we review recent progress on the rapidly growing field of restriction factors, focusing on the antiretroviral activities of APOBEC3G, TRIM5, tetherin, SAMHD1, MOV10, and cellular microRNAs (miRNAs), and the counter-activities of Vif, Vpu, Vpr, Vpx, and Nef.

## Review

As an obligatory intracellular parasite with limited genome size, retroviruses interact with both supportive and inhibitory host factors to complete their life cycle. Supportive factors help the virus enter the cell, duplicate its viral genome, make viral proteins, and release new progeny particles, while inhibitory factors could, in principle, intervene against the virus at every step of replication. These inhibitory factors are collectively called host restriction factors. Unique from other viruses, retroviruses require replication steps such as RNA reverse transcription and DNA integration, which create additional targets for restriction. Historically, the first host restriction for retroviruses was discovered when murine leukemia virus (MLV) infection was found to be inhibited by the Friend virus susceptibility factor-1 (Fv1) [1]. In the mouse genome, there are at least two *Fv1* alleles (*Fv1*^*n*^, *Fv1*^*b*^) that confer resistance to B-tropic MLV (B-MLV) or N-tropic MLV (N-MLV) infection. The B-MLV strains efficiently infect *Fv1*^*b/b*^ homozygous Balb/c mice but not the *Fv1*^*n/n*^ homozygous NIH/Swiss mice, whereas the N-MLV strains have an opposite tropism. The *Fv1* gene is located on mouse chromosome 4 [2], which encodes an endogenous retrovirus Gag-like protein [3]. Fv1 recognizes the MLV capsid (CA) protein through a single residue at position 110 [4], and blocks the nuclear import of reverse transcribed retroviral pre-integration complex [5], but the precise mechanism is still unknown.

**Figure 1:**
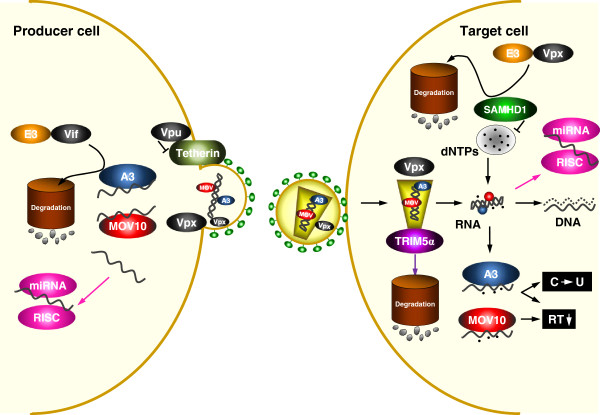
**Schematic illustration of the action of host restriction factors during primate lentivirus replication.** In viral producer cells, both A3 and MOV10 proteins are packaged into virions via interaction with Gag and RNA. A3 proteins can be targeted by Vif for proteasomal degradation, and viral RNAs can be targeted by specific microRNAs (miRNAs) for suppression. Vpx is also packaged into virion via direct interaction with Gag. In addition, viral release can be inhibited by the cell surface protein tetherin, but it is counteracted by Vpu or Nef (not shown). In target cells, TRIM5α interacts with incoming Gag proteins and triggers premature viral uncoating, resulting in inhibition of viral reverse transcription and nuclear import. Reverse transcription can also be directly inhibited by MOV10 and A3 proteins, or indirectly by SAMHD1 after depleting intracellular dNTP pool. However, SAMHD1 can be neutralized by Vpx through proteasomal degradation. In addition, A3 proteins catalyze C-to-U cytidine deamination on newly synthesized viral cDNA, and viral RNAs can be targeted by miRNAs, which also result in inhibition of viral replication.

The initial observations on Fv1 have been followed by the discovery of additional restriction factors in mammalian cells [6]. In general, restriction factors have been identified from non-permissive cells, where virus replication is severely restricted. The restriction phenotype can be dominant and potent and can suppress viral replication up to several orders of magnitude. Thus, when a non-permissive cell is fused with a permissive cell, the heterokaryon inherits the restrictive phenotype. Another finding with restriction factors is that they can display hallmarks of positive genetic selection during evolution, indicative of their beneficial advantage to the host in settings of host-pathogen conflicts. Moreover, restriction factors can be constitutively or inducibly (e.g. induced by interferon) expressed, and many viruses have evolved countervailing stratagems to neutralize the activities of restriction factors. For example, HIV-1 co-opts the host ubiquitin/proteasome system (UPS) to degrade cellular restriction factors. The recent advances accrued from studying restriction factors have expanded our views on virus-host interaction as well as host innate immunity to viral infections [7]. The findings have not only provided a more comprehensive understanding of the virus life cycle, but have also offered clues on new antiviral mechanisms and targets. Below, we survey the antiviral activities of several cellular restriction factors that impede HIV replication, including APOBEC3, TRIM5α, tetherin, SAMHD1, MOV10 and cellular miRNAs; and we outline viral countermeasures to subdue these restrictions (Figure 1).

### APOBEC3 proteins

**Figure 2:**
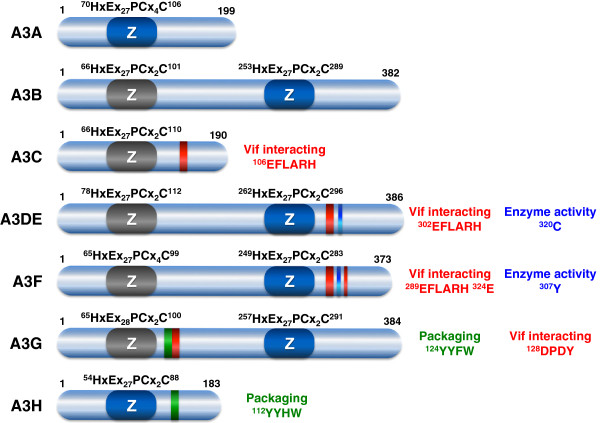
**Schematic illustration of seven human A3 proteins.** Numbers indicate amino acid positions. The zinc-coordinating cytidine deaminase (Z) domain motifs are indicated. The enzymatically active Z domain is presented in dark blue, and the inactive Z domain is in grey. The Vif-interacting motif (red), virion packaging motif (green), and critical residues affecting the enzymatic activity (blue) are all indicated.

APOBEC3 (A3) refers to Apolipoprotein B mRNA-editing enzyme catalytic polypeptide-like 3 proteins, which include A3A, A3B, A3C, A3DE, A3F, A3G, and A3H. These genes are tandemly arrayed on human chromosome 22 and were discovered by homology search using the APOBEC1 signature sequence [8,9]. The *A3DE* gene was initially proposed as two separated genes (*A3D*, *A3E*); later, it was shown to produce alternatively spliced mRNAs from a single gene. Unlike humans, the mouse genome encodes only a single *A3* gene. Altogether these proteins belong to the cytidine deaminase family that includes four additional members: activation-induced cytidine deaminase (AID), APOBEC1, APOBEC2, and APOBEC4. All APOBEC proteins have one or two copies of zinc-coordinating deaminase domain (the Z domain) with a signature motif HxEx_23–28_PCxxC, which catalyzes cytidine deamination that converts cytosines to uracils (C to U) on a variety of vertebrate-specific RNA/DNA targets (Figure 2). APOBEC1 regulates lipid metabolism [10], and AID contributes to antibody production [11]. The physiological function of APOBEC2 and APOBEC4 is still unknown. A3A, A3C, and A3H have one copy of Z domain, whereas A3B, A3DE, A3F, and A3G have two. The functions of these A3 proteins on viral infection were not clear, until it was discovered that A3G has a very potent anti-HIV-1 activity.

### Discovery of A3 protein antiretroviral activity

The discovery of A3’s antiviral role originated from the characterization of one HIV-1 accessory gene function. The *vif* gene encodes a ~23 kDa “virion infectivity factor”, which is expressed in all lentiviruses except for equine infectious anemia virus (EIAV). Vif is absolutely required for HIV-1 replication in non-permissive cells, although it is not required in permissive cells [12,13]. Vif must be present in the viral producer cells; otherwise, viral replication is strongly blocked at the reverse transcription step in target cells [13]. It was hypothesized that permissive cells either express a Vif-like positive factor, which would replace the *vif* gene function, or that non-permissive cells express an inhibitory factor, which would require Vif for counteraction [14]. A genetic complementation assay performed by fusing the non-permissive cells with permissive cells demonstrated that the non-permissive phenotype is inheritable, indicating that the non-permissive cells express a dominant inhibitory factor [15,16]. By subtraction of cDNAs of non-permissive cells from those of permissive cells, this inhibitor was identified as A3G [17].

The discovery of A3G’s anti-HIV-1 activity has invited further investigation on its sister proteins. A3F was identified as the second restriction factor from this family [18,19], and the inhibitory activities of A3B, A3DE, and A3H were subsequently uncovered [20-23]. Among these five anti-HIV A3 proteins, A3B is expressed very poorly in primary tissues due to unexplained reason [24], and the *A3H* gene is highly polymorphic [25-27]. So far, seven A3H haplotypes (I-VII) have been identified in human populations [26,28]. However, only haplotypes II, V, and VII could be stably expressed, and their total allelic frequencies are less than 30%. In addition, due to a Tyr-to-Cys substitution at position 320, the human *A3DE* anti-HIV-1 activity is reduced by approximately 20-fold [29], although the potent A3DE antiviral activity is still retained in chimpanzees [30]. The endogenous A3DE, A3F, and A3G have the most relevant anti-HIV-1 activity *in vivo*[31], although A3DE and A3F may have weaker activity than A3G [32].

The antiviral activity of A3 proteins extends to other retroviruses such as hepatitis B virus (HBV) [33], SIV [34], HTLV-1 [35], foamy virus [36-38], EIAV [39], MLV [40], and mouse mammary tumor virus (MMTV) [41]. While A3A and A3C do not have anti-HIV-1 activity, A3A strongly inhibits adeno-associated virus (AAV) replication [42], and A3C inhibits SIV replication [43]. In addition, all the A3 proteins inhibit the replication of retrotransposons, although the levels of this activity may vary [44]. *A3* expression is highly inducible by type I interferons (IFNs), particularly in myeloid-derived cells [45,46]. Both A3DE and A3G genes have been subjected to strong positive selection during evolution [30,47]. In addition, A3 knockout mice are more susceptible to MMTV, MLV, and mAIDS virus infection [41,48-50]. Thus, *A3* genes play an important role in defending *in vivo* against both exogenous retrovirus and endogenous retroelements.

### Action of A3: deamination-dependent and -independent inhibition

To inhibit viral replication, A3 proteins are typically packaged into retroviral particles from the producer cells [51], and delivered to the target cells by infection (Figure 1). The human A3G has 384 amino acids, and contains duplicative Z domains with different functions (Figure 2). Unlike the C-terminal Z domain, the N-terminal Z domain is a pseudocatalytic domain that does not have any enzymatic activity. However, the N-terminal Z domain has high affinity for RNA-binding and determines A3G packaging into virions [52]. In conjunction with an adjacent ^124^YYxW^127^ motif [53], the pseudocatalytic site of the N-terminal Z domain interacts with the HIV-1 Gag protein in the nucleocapsid (NC) region, in an RNA-dependent manner [54-61], resulting in A3G packaging into HIV-1 virions. The YYxW motif is also present in other A3 proteins, and it is required for A3H packaging [28], indicating its important role in regulating this critical step of A3 antiviral activity.

After being delivered to target cells, the C-terminal enzymatically active Z domain inhibits viral replication by either cytidine deamination-dependent or -independent mechanisms at the reverse transcription step. The active Z domain can directly deaminate cytosines (C) to form uracils on newly synthesized minus-strand viral cDNAs, leading to changes in the viral sequences [39,62-64]. Since these edited cDNAs contain uracils that are usually not present in DNA molecules, they may be recognized by DNA repair enzymes for degradation. Otherwise, they are copied into the plus strand DNA during DNA synthesis, generating DNA molecules containing G-to-A hypermutations that compromise viral genome integrity. This process is generally termed the cytidine deamination-dependent mechanism. Not every cytosine is mutated, and different A3 proteins have their own dinucleotide preference sequence. For example, A3G prefers to mutate minus strand 5’-CC-to-CU and cause plus strand 5’-GG-to-AG mutation [65]; A3B, A3DE, A3F, and A3H mutate 5’-TC-to-TU and cause 5’-GA-to-AA mutation [22-24,65]; and A3DE also mutates 5’-CG-to-UG and causes 5’-GC-to-AC mutation [22]. All these three types of G-to-A hypermutations are detectable from HIV-1 patients, and it is still unclear which enzyme takes the major responsibility for the 5’-GT-to-AT mutation [66]. The three-dimensional structure of the A3G C-terminal domain has been solved by nuclear magnetic resonance (NMR) and X-ray crystallography [67-69]. This part of the protein shows a globular structure that is formed by five β-sheets and six α-helices and contains a substrate-binding surface. The X-ray structure further shows a deep groove in this region for substrate binding, which is composed of several critical residues including N244, W285, R313, Y315, D316, and R374. The Y315 residue (or Y307 in A3F) is crucial for deaminase activity. This residue is replaced with a Cys (C320) in human A3DE (Figure 2); this change significantly reduces A3DE anti-HIV-1 activity [29]. Taken together, the findings provide strong evidence that deaminase activity is important to A3’s anti-HIV-1 activity.

In addition to the introduction of catastrophic mutations, A3F and A3G directly block the process of viral reverse transcription. They reduce the efficiency of tRNA^lys3^ priming to the viral RNA template, elongation of reverse transcription, and DNA strand transfer [70-76]. Moreover, they block viral integration [75,77]. All these inhibitory mechanisms are still not fully understood, but they are generally thought to arise from a deamination-independent antiviral mechanism. Notably, there is a great deal of confusion in the literature regarding deaminase-dependent versus deamination-independent mechanisms. Although initial investigations suggested that the deaminase activity was not required for antiviral activity, later studies demonstrated that it is required when the A3 proteins are expressed at physiological levels [78-81]. In contrast, it has been consistently observed that A3 proteins block viral replication even in the absence of cytidine deamination, particularly when HTLV-1, AAV, HBV, and retrotransposons are targeted by A3 proteins [33,35,42,82-87]. Thus, the deaminase activity is always required, and in various settings the mechanism is deamination-independent.

Although a different opinion exists [88], several lines of evidence indicate that A3 proteins may require other cellular cofactors. First, APOBEC1, which is the founding member of this family, requires a cofactor. APOBEC1 introduces a premature stop codon in apolipoprotein (apo) B100 mRNA through C-to-U editing to produce a truncated form of this protein [10]. This process requires an interaction with the APOBEC1 complementation factor (ACF) to form a holoenzyme, or so-called editosome to edit the target sequence [89,90]. Thus, A3 proteins may also require a cellular cofactor for target recognition. Second, both A3F and A3G form two distinctive protein complexes: a high molecular mass (HMM) complex over 700 kDa and a low molecular mass (LMM) complex below 100 kDa [91]. The A3G HMM complex contains cellular RNAs and predominantly exists in immortalized cell lines; this complex changes into an enzymatically active LMM complex when treated with RNases. Over a hundred A3G-binding proteins, most of which are RNA-binding proteins, have been identified from these complexes [92-94]. It is conceivable that some of them may facilitate A3G virion packaging and/or antiviral activity. Third, the cellular expression levels of A3G do not always correlate with its antiviral activity. This phenomenon is particularly remarkable in the human CD4^+^ CEM-derived T cells. The parental CEM cell line is non-permissive for Vif-deficient (ΔVif) HIV-1 due to A3G expression, and its derivative CEM-SS cells are permissive because they do not express A3G. Notably, its derivative A3.01 and CEM-T4 cell lines express significant levels of A3G, but they can support ΔVif HIV-1 replication [79]. Absence of a critical cofactor may explain why A3 proteins are not active in these cells.

### Action of Vif: degradation-dependent and -independent inactivation

Because virion packaging is required for A3G antiviral activity, a critical action of Vif is to exclude A3 proteins from virions in order to protect viral replication [95]. This is achieved by degradation-dependent and/or independent mechanism in the viral producer cells.

**Figure 3:**
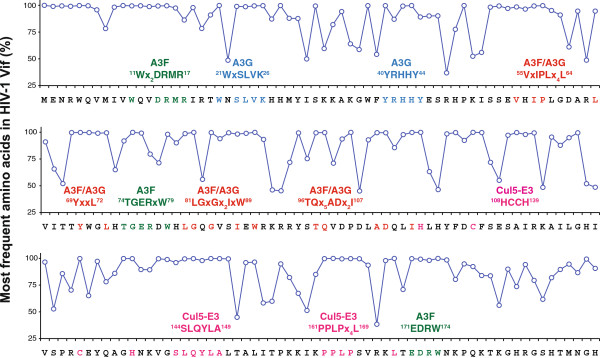
**Amino acid sequence homology of known HIV-1 Vif functional motifs.** Numbers indicate amino acid positions. At each position, the most common amino acid is identified and plotted as a percentage of all amino acids at that position. Motifs that regulate A3F binding are in green; those that regulate A3G binding are in blue; those that regulate both A3G and A3F binding are in red; and those that regulate Cul5/EloBC binding are in pink.

The degradation-dependent mechanism hijacks the cellular proteasomal pathway to degrade A3 proteins to ensure that insufficient A3 proteins are packaged into the virions. HIV-1 Vif has 192 amino acids and contains 12 highly conserved motifs (Figure 3). These motifs form discontinuous surfaces, so that Vif can interact with A3 and E3 ligase. First, Vif interacts with the Cul5 E3 ubiquitin ligase complex, which includes Cullin 5 (Cul5), Elongin B (EloB), and Elongin C (EloC) [96]. This interaction is mediated by its three C-terminal motifs. The ^108^Hx_5_Cx_17-18_Cx_3-5_H^139^ motif, which is also called the HCCH zinc finger, binds to Cul5 [97-99]; the ^144^SLQYLA^149^ motif, which is also called the BC-box, binds to EloC [100,101]; and the ^161^PPLPx_4_L^169^ motif, which is also called the Cul box, binds to Cul5 [101,102]. The ^161^PPLP^164^ subdomain has multiple activities, which not only determine Vif dimerization [103], but also regulate Vif binding to A3G [104-106] and EloB [107]. Second, Vif interacts with its target A3DE, A3F, and A3G via other widely distributed motifs. The ^21^WxSLVK^26^[108,109] and ^40^YRHHY^44^[110] motifs regulate Vif binding to A3G; the ^11^Wx_2_DRMR^17^[110], ^74^TGERxW^79^[111], and ^171^EDRW^174^[112] motifs regulate Vif binding to A3F; and ^55^VxIPLx_4_L^64^[111], ^69^YxxL^72^[113], ^81^LGxGx_2_IxW^89^[112], and ^96^TQx_5_ADx_2_I^107^[114] motifs regulate Vif binding to both A3G and A3F. Those motifs that regulate Vif interaction with A3F also regulate Vif interaction with A3DE [115].

The Vif-interactive domains on A3DE, A3F and A3G have also been identified. Although A3F and A3G share 50% amino acid sequence identity, they use different domains to interact with HIV-1 Vif. Vif binds to the A3G N-terminal subdomain ^128^DPD^130^[53], and it recognizes the A3F C-terminal ^289^EFLARH^294^ domain and a residue ^324^E [116-118]. The EFLARH domain, also present in A3C and A3DE, further determines their Vif sensitivity (Figure 2) [118]. These interactions assemble the A3-Vif-Cul5 E3 protein complex, which induces polyubiquitylation and proteasomal degradation of these A3 proteins.

The A3G polyubiquitylation sites have been mapped to lysine residues 297, 301, 303, and 334 [119]. However, there is evidence that A3G is polyubiquitylated through the N-terminus [120,121]. Notably, Vif itself is polyubiquitylated by the same E3 ligase [100] and is degraded via the proteasome [122-124]; and Vif polyubiquitylation is very critical for A3G proteasomal degradation [125]. To initiate proteasomal degradation, a protein should have at least two signals: an attached polyubiquitin chain and an unstructured region (USR) [126]. The polyubiquitin chain is required for proteasome recognition, and the USR allows the protein to enter the narrow proteasome entrance channel to initiate degradation. Although both signals can be present in the same protein, they can also work in *trans* in two different proteins that bind to each other. A protein missing any signal may remain stable, until it binds to an adaptor protein to compensate for the missing signal. Since A3G is well structured, it is possible that A3G degradation is dependent on the polyubiquitin chain on Vif, but not on A3G itself. Thus, more investigation is needed to understand better whether A3G polyubiquitylation is critical for its neutralization, and how Vif polyubiquitylation contributes to this process.

The action of Vif is highly species-specific. Vif from HIV-1 only inactivates A3G from human; and Vif from SIV, isolated from African green monkey (AGM), does not inactivate human A3G. Nevertheless, Vif from SIV isolated from rhesus macaque (MAC) inactivates A3G from all humans, African Green monkeys, and macaques [127]. The resistance of agmA3G to HIV-1 Vif is due to a single mutation in the ^128^DPD^130^ motif of A3G, which is adjacent to A3G ^124^YYxW^127^ packaging motif [53]; notably, this motif is changed to ^128^KPD^130^ in the agmA3G [128-130]. In addition, a N-terminal domain in HIV-1 Vif, ^14^DRMR^17^, determines Vif activity for A3G from different species [131].

Recently, the core-binding factor β (CBF-β) was identified as a critical cofactor for Vif [132,133]. CBF-β forms a heterodimer with the RUNX transcription factors and increases the complex binding to the target DNA [134]. Knockdown of CBF-β expression in 293T cells was found to compromise Vif’s ability to trigger A3G degradation, but it is still controversial whether CBF-β stabilizes Vif protein itself, or simply induces Vif conformational changes to stabilize the Vif-Cul5 complex. In addition, its critical role in Vif function needs to be verified in human T cells.

Many extant observations can be explained by the A3G degradation-dependent inhibition model; however, emerging evidence suggests that this is not the only mechanism for Vif to neutralize A3G. For example, if Vif-induced A3G degradation is fully responsible for A3G inactivation, then the levels of A3G reduction by Vif in viral producer cells should be proportional to those in virions. In fact, the levels of A3G reduction by Vif in virions are always much more pronounced than those in viral producer cells. Thus, Vif is apparently able to block A3G encapsidation in the absence of an induction of degradation [135]. The existence of this degradation-independent mechanism is supported by two mutational studies. The A3G C97A mutant is resistant to Vif induced proteasomal degradation, but its activity is still neutralized by Vif [136]. In addition, Vif proteins that show different ability to degrade A3G exhibit similar efficiencies in neutralizing A3G [137]. In fact, Vif can inactivate A3G enzymatic activity in *E. coli*, which does not have an UPS for protein degradation [138]. Thus, it seems reasonable that Vif employs both degradation-dependent and -independent mechanisms to counteract A3G’s antiviral activity.

## A3 and HIV evolution

Because HIV-1 replicates in A3-expressing cells in the presence of Vif, it has been thought that Vif completely counteracts A3’s antiviral activity. However, the virus may not benefit from a complete inhibition of A3 activity. Although lethal mutations inhibit viral replication, sublethal mutations can promote viral propagation in hosts through a promotion of genome evolution. HIV-1 is notorious for its rapid evolution. It maintains an optimal mutation rate that allows viral escape from adaptive immunity and development of drug resistance. Among various mutations, the appearance of G-to-A hypermutations is the most frequent [139], which may explain why the HIV-1 genome is extremely A-rich [140,141]. Although G-to-A hypermutations have been attributed to the errors of the low-fidelity viral reverse transcriptase and viral genomic recombination, it has been recently appreciated that A3 proteins are significantly responsible for this type of mutation. Analyses of viral hypermutations at population levels have detected both GG→AG or GA→AA dinucleotide motifs for introducing hypermutations [142-145]. In addition to inhibition of viral replication, A3 proteins may create beneficial hypermutations, which generate viral quasispecies and diversify viral genomes [146-148]. Both A3G and A3F facilitate viral adaptation to the new drug treatment environment if their activities are not completely neutralized by Vif [149,150]. Several mechanisms may cause A3 activity to not be completely neutralized by Vif. First, the relative levels of protein expression may determine neutralization. Because A3 expression is interferon inducible, A3 expression level may well exceed the Vif expression level at the early stage of viral replication, which may make it impossible for Vif to neutralize fully A3 proteins. Second, the degree of neutralization may be regulated at the levels of protein quality. The *vif* gene itself could become the target for hypermutation and become polymorphic during natural infection. Any mutation that interferes with Vif binding to A3 and/or Cul5 E3 ligase will compromise A3 neutralization by Vif, resulting in incomplete A3 inactivation [151,152]. Thus, it is anticipated that a balance between Vif and A3 proteins has to be established over time to maintain optimal viral fitness and composition of viral quasispecies *in vivo*. It is also conceivable that viral replication could be inhibited if this balance is disrupted by pharmacological intervention [153], either by a complete inhibition of Vif activity [154-156], or by a commensurate inhibition of A3 enzymatic activity [157].

### TRIM5

**Figure 4:**
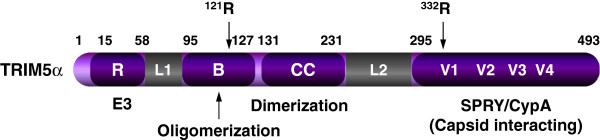
**Schematic illustration of human TRIM5α protein.** Numbers indicate amino acid positions. The RING finger (R), B-box (B), coiled-coil motif (CC), two linkers (L1, L2), and SPRY domain are indicated. Four variable regions in SPRY, the critical residue (R121) in the B domain that determines oligomerization, and the critical residue (R332) in V1 region that determines species-specific Gag binding are also indicated.

TRIM5 belongs to the tripartite motif (TRIM)-containing protein family, which has over 100 members [158]. All TRIM proteins have three motifs, including a N-terminal RING finger motif, followed by one or two B-box motifs, and then by a coiled-coil motif [159-161]. The RING, B-box, and coiled-coil motifs are also collectively called the RBCC domain. The C-terminal regions of these proteins vary, but most of them have a SPRY (also known as B30.2) motif. The RING finger binds to two zinc atoms, and usually has E3 ubiquitin ligase activity; the B-box and CC domains promote protein oligomerization. Human TRIM5 has six major isoforms, with the α isoform most abundantly expressed (~50%) [162]. Human TRIM5α has 493 amino acids, and it is the only isoform that has the C-terminal SPRY domain (Figure 4). Its RING and B-box 2 domains are separated by the linker 1 (L1) region, and its coiled-coil and SPRY domains are separated by the linker-2 (L2) region. The SPRY domain has been functionally replaced with another host protein cyclophilin A (CypA) in a number of monkey species, resulting in another protein TRIM-CypA [163]. TRIM5α and TRIM-Cyp are the only isoforms that have antiretroviral activity and inhibit retroviral replication in a species-specific manner.

### Discovery of TRIM5 antiretroviral activity

Although Fv1 is only expressed in mice, the N-MLV strains encounter an Fv1-like restriction in non-murine species including humans, and this unknown MLV inhibitor was named restriction factor 1 (Ref-1) [164]. Ref1 also inhibits EIAV replication in human cells [165]. HIV-1 and some SIV strains encounter another Fv1-like restriction when they infect some non-human species. For example, HIV-1 replication is inhibited in the Old World Monkeys (rhesus macaques, African green monkeys) and New World Monkeys (squirrel monkeys, common marmosets); SIV (SIVmac) infection is blocked in the squirrel monkeys. The unknown HIV-1 and SIV inhibitors were named lentivirus susceptibility factor 1 (Lv1) [166,167]. Fv-1, Ref-1, and Lv-1 share remarkable similarities in their viral inhibition. First, they all target an early post-entry step. Both Ref-1 and Lv-1 act at steps before or after reverse transcription, whereas Fv1 acts at a step after reverse transcription but before integration. Second, they all target viral CA proteins. The same single CA 110 residue that differentiates between N- and B-tropism in mice also determines MLV tropism in human cells. Similarly, HIV-1 and SIVmac restriction in some primate cell lines is determined by sequences within the CA-p2 region of Gag [167-169]. Third, all these restrictions can be released by a high multiplicity of infection (m.o.i.), indicating that they are saturable. In due course, the Lv1 restriction activity was first identified as the TRIM5α protein from rhesus monkey cells and later as the TRIM-Cyp fusion from the owl monkey cells [170,171]. Subsequently, Ref-1 was identified as the human TRIM5α protein [172]. Thus, Ref-1 and Lv1 are specifies-specific TRIM5α proteins that have different activities against different retroviruses.

### TRIM5 E3 ubiquitin ligase activity

The TRIM5 RING finger motif features a cysteine-rich consensus that contains two interleaved Zinc-binding sites. This motif may simultaneously bind ubiquitination enzymes and their substrates, and hence exhibits an E3 ligase activity. For example, the RING-box-1 (Rbx1) is an essential component of the Skp1-cullin1-F box (SCF) complex, which is a multi-protein E3 ubiquitin ligase that regulates cell cycle [173]. Human TRIM5 has a ββα RING fold with a putative E2-binding region [174]. The TRIM5 E3 ligase activity was first demonstrated in TRIM5σ [175], and recent studies suggest that this activity may contribute to the restriction activity (see below) [176,177].

TRIM5α triggers self-polyubiquitylation using UbcH5 as an E2-conjugating enzyme [178], but the role of this self-polyubiquitylation is still not completely clear. TRIM5α is relatively unstable in cells, with a protein half-life only about 50 to 60 minutes; but current data suggest that the proteasomal pathway is not responsible for its rapid turnover [178] and that this rapid turnover is not required for TRIM5α’s antiretroviral activity [179]. Nevertheless, the TRIM5α turnover is further enhanced when cells are infected with restriction-sensitive viruses. In this setting, the proteasomal pathway is responsible for its enhanced degradation [180] and TRIM5α has been found to be associated with proteasomal subunits in cells [181]. Interestingly, the inhibition of the proteasomal pathway does not significantly disrupt overall TRIM5α restriction, despite disrupting TRIM5α-mediated inhibition of viral reverse transcription [182]. Accordingly, two independent inhibitory mechanisms have been proposed. The first is accelerated viral uncoating by TRIM5α interaction with CA, which triggers premature uncoating and proteasomal degradation of the viral reverse transcription complex. The second is a block to the nuclear translocation of the viral preintegration complexes, which has also been described for Fv1 [182].

In addition to a direct inhibition of viral replication, TRIM5α can be a signaling molecule that activates the NF-κB pathway [183]; this pathway is shared in several receptor-signaling routes including those used by Toll-like receptors (TLRs). Stimulation of TLRs induces autophosphorylaton and activation of TGF-activated kinase 1 (TAK1), and TAK1 in turn activates IκB kinase, leading to the activation of NF-κB. TAK1 can be directly activated by unanchored (free C-terminus) K63 polyubiquitin chains [184]. In fact, activated TRIM5α interacts with UBC13 and UEV1A E2 enzymes and triggers the production of unanchored K63-linked ubiquitin chain, resulting in the activation of TAK1 [176], and this activity also extends to several other TRIM family members [185]. In addition, TRIM5α was recently reported to traffic through the nucleus [186], which may be related to this activity. Thus, by interacting with viral cores, TRIM5α induces TAK1 autophosphorylation and activates the NF-κB pathway, which indirectly defends against viral infection via signaling through the innate pattern-recognition receptor-mediated immune responses.

The antiviral activity of TRIM5α can be disrupted by treatment with As_2_O_3_ (arsenic trioxide), but the mechanism for this effect is still unclear [187,188]. Trivalent arsenic (As^III^) has very high affinity for free thiols, which are present in the Cys residues in TRIM5α. As^III^ has very broad biological activities, which are largely mediated by direct interaction with Cysteines in target proteins [189]. When these Cys residues are located in close proximity, this interaction results in their cross-linking, which causes protein conformational changes through S-As-S bond formation. As^III^-induced conformational changes have different impacts on protein function. Notably, As^III^ directly binds to the Cys residues in the RING motif of TRIM19, which is also known as promyelocytic leukemia (PML) protein, and this interaction makes PML more accessible to SUMOylation, resulting in enhanced PML degradation [190]. It remains to be determined whether As^III^ disrupts TRIM5α’s antiviral activity by a similar mechanism.

### TRIM5 oligomerization

Unlike the RING domain, the B-box 2, coiled-coil, and SPRY domains are absolutely required for TRIM5α’s antiretroviral activity [191,192]. TRIM proteins share a common feature in supporting the structure of various cytoplasmic and nuclear bodies through self-association [160]. TRIM5α proteins are found in cytoplasmic bodies, although these cytoplasmic structures do not directly contribute to antiviral activity. TRIM5α oligomerizes at two different levels, which are determined by the B-box 2 and coiled-coil domains. The coiled-coil domain determines TRIM5α dimerization, which is a lower-order oligomerization [193,194]; the B-box 2 domain, particularly the residue R121, triggers a higher-order oligomerization through association of these dimers (Figure 4) [195]. Dimerization is essential for higher-order oligomerization, and the RING domain and linker 2 region are also involved in the oligomerization process [196]. HIV-1 has a cone-shaped viral core, which is supported by CA proteins that form pentagonal and hexagonal lattices, where the CA N-terminal domain (NTD) forms either hexameric or pentameric rings, and the C-terminal domain (CTD) forms symmetric homodimers that connect the rings into lattices [197]. TRIM5 proteins spontaneously assemble into hexagonal lattices, which match the symmetry of CA lattices, and this assembly can be enhanced by recombinant CA proteins that have already preformed the conical structure [198,199]. Thus, the higher-order TRIM5α structure increases its binding for the viral CA protein.

As introduced earlier, TRIM5α accelerates viral uncoating to block viral replication. Uncoating occurs within several hours after viral entry, which releases the viral genome from the viral core by removing capsid and envelope [200]. After HIV-1 entry, the CA proteins are detectable in the cytosolic fraction as both pelletable and soluble forms. The pelletable form could be derived from the intact cores that have not been uncoated, and the soluble form could be derived from uncoated cores. TRIM5α accelerates the conversion of the viral CA proteins from pelletable to soluble forms [201]. Moreover, incubation of the preassembled CA recombinant proteins with TRIM5α resulted in the disruption of the CA conical structure, likely by weakening the CA CTD-CTD interfaces between hexamers [202]. Thus, it is suggested that TRIM5α could form the hexagonal structures on top of the capsid lattices, which disrupts the core and then further destruction of viral proteins could occur via the proteasomal machinery. In this model, a linkage exists between TRIM5α degradation by the proteasome and accelerated viral uncoating by TRIM5α. Since TRIM5α is associated with the proteasomal machinery [181] and its contribution to uncoating is blocked by proteasomal inhibitors, proteasomes should be engaged in TRIM5α enhancement of core disassembly.

### TRIM5 cross-species activity

Unlike other restriction factors, TRIM5α normally does not inhibit retroviruses isolated from the same host species. For example, human TRIM5α (hsTRIM5α) has a very weak activity for HIV-1, but it strongly inhibits EIAV and N-MLV; TRIM5α from rhesus monkeys (rhTRIM5α) does not inhibit the SIVmac strains, but it strongly inhibits HIV-1 and some other SIV strains [171]. However, a single residue exchange in the hsTRIM5α SPRY domain (R332) with the corresponding rhTRIM5α residue (P334) is sufficient to lead to HIV-1 restriction by the altered hsTRIM5α [203,204]. This residue is among a cluster of residues, which are located in the first variable region (V1) and have been found to be under strong positive selection (Figure 4) [205]. Analysis of TRIM5α sequences from different species identified four variable regions (V1, V2, V3, V4) in the SPRY domain [203,206], and three of them (V1, V2, V3) contribute to TRIM5α specificity of retrovirus restriction [191]. The rhesus macaque *TRIM5* gene is particularly polymorphic in the SPRY region. A three-residue replacement with another residue in the V1 region results in TFP/Q polymorphisms, and in some cases, the entire SPRY region is replaced with the *CypA* gene to produce a new TRIM-CypA protein. Accordingly, the *TRIM5* gene is classified into three allelic groups based on the SPRY domain in the various species: *TRIM5*^*CypA*^, *TRIM5*^*TFP*^, and *TRIM5*^*Q*^[207]. These polymorphisms have significant impact on SIV cross-species infection [207-209]. Consistent with host specificity, a large population study has found that common human variants of TRIM5α has little to no effect on HIV-1 disease progression [210], suggesting that the role of this protein in the human genome is not to mitigate HIV-1 infection and instead serves a not yet understood function.

Although the SPRY domain is not required for hexagonal array formation, compelling evidence suggests that this domain is directly engaged in the interaction with CA proteins; and this interaction specifies cross-species restriction activity [188,201]. Detection of direct TRIM5α-CA interaction has been difficult, because TRIM5α does not bind to monomeric or soluble CA proteins [211]. This interaction only becomes detectable in a SPRY-dependent manner when TRIM5α is directly incubated with purified viral cores or pre-assembled recombinant CA proteins [188,201]. These results suggest that TRIM5α recognizes CA proteins in a conformation-dependent manner, which is consistent with a previous finding that only stable and mature cores could neutralize the TRIM5α antiviral activity [212]. A structural analysis confirmed the direct interaction between the SPRV domain and CA protein, and that the variable regions, particularly the V1 region, are responsible for this interaction [213]. This study also uncovered that a single-site SPRY-CA binding is weak, and that optimal interaction involves multiple CA epitopes, which may explain why multivalent binding within the spacing of CA lattice is required for viral restriction.

### Tetherin

Tetherin was originally identified (and termed HM1.24, BST-2, or CD317) as a specific cell surface marker of terminally differentiated B-cells, through screening of mouse monoclonal antibodies raised against the human plasma cells [214]. The gene encoding this protein was independently cloned from the human rheumatoid arthritis-derived synovial cells and termed BST-2 [215]. The protein was proposed to potentially serve as a target antigen for the immunotherapy of multiple myeloma since its mouse and humanized monoclonal antibodies showed anti-tumor activity with antibody-dependent cellular cytotoxicity both *in vitro* and *in vivo*[216,217]. The protein, re-designated as CD317, was then found to be highly expressed in B cells at all differentiation stages, and in bone-marrow CD34^+^ cells, and in T cells [218].

**Figure 5:**
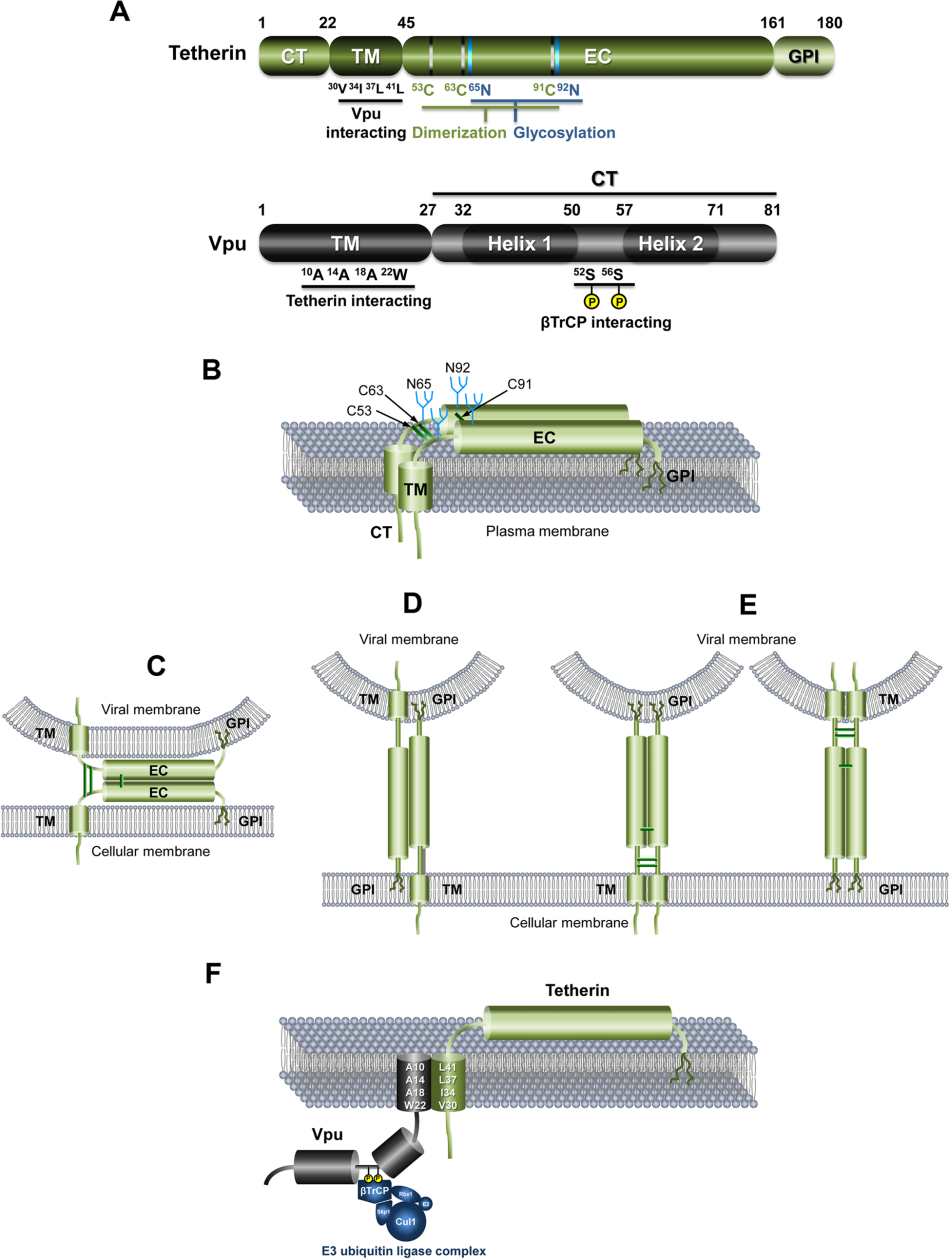
**Configuration models of human tetherin.** (**A**) Schematic illustrations of tetherin and Vpu. Numbers indicate amino acid positions. Critical residues of each protein are indicated. (**B**) Structure of tetherin. Tetherin comprises a short amino-terminal cytoplasmic tail (CT), followed by an α-helical transmembrane (TM) domain and a coiled-coil extracellular (EC) domain that is linked back to the plasma membrane by a carboxy-terminal glycophosphatidylinositol (GPI) anchor. The EC domain contains N-glycosylation sites and cysteine residues involved in disulfide-bond formation. (**C-F**) Configuration models of tetherin. (**C**) The EC self-interaction model. Individual tetherin monomers are anchored at both ends to the same membrane, with interaction between the ECs of cell-bound and virion-bound monomers. (**D**) Anti-parallel membrane-spanning model. Monomers are anchored in both membranes with opposing orientations. (**E**) Parallel membrane-spanning model. Monomers are anchored in both membranes with the same orientation. (**F**) HIV-1 Vpu and tetherin interact through their TM domains. Key amino acids involved in the interaction are depicted in the TM helices. Interaction of Vpu’s CT with the E3 ubiquitin (Ub) ligase via the βTrCP subunit is required for Vpu-induced tetherin down-regulation.

Tetherin is an interferon (IFN)-inducible type II membrane protein, consisting of a short amino-terminal cytoplasmic tail (CT) followed by an α-helical transmembrane (TM) domain, a coiled-coil extracellular (EC) domain, and a carboxy-terminal glycophosphatidylinositol (GPI) component that acts as a second membrane anchor [219] (Figure 5A and 5B). This double-anchored form determined by the TM and the GPI anchor is unusual and only shared with a minor isoform of the prion protein [220]. Three cysteine residues located at the EC domain form intermolecular disulfide bonds [221], resulting in homodimerization of the protein [222]. At the cell surface, tetherin is located in cholesterol-rich lipid microdomains, also termed lipid rafts, through the GPI anchor. The TM domain resides outside the lipid rafts [219], placing the CT in a suitable position to indirectly interact with the actin cytoskeleton [223]. Tetherin is physiologically endocytosed from lipid rafts in a clathrin-dependent manner through interaction with α-adaptin of the AP-2 complex [224].

### Discovery of tetherin’s antiviral activity

Early studies on HIV-1 replication showed that the accessory viral protein U (Vpu) was required for efficient viral particle release in a cell-type dependent manner [225,226]. This led to the hypothesis that the requirement was due to either the existence of unknown restriction factor(s) or the lack of cofactor(s) in non-permissive cells. Some years later, experiments generating heterokaryons between permissive and non-permissive cells provided the answer [227]; the latter cells likely express restriction factor(s) that could be counteracted by Vpu [228], exactly like Vif-responsive cells express A3G that is counteracted by Vif. It was then shown that Vpu-deleted HIV-1 particles captured at the cell surface were detached by treatment with subtilisin protease, implying that the endogenously expressed host restriction factor is a membrane-associated cell-surface protein [229]. Soon afterwards, this endogenous factor was found to be IFN α-inducible and indeed its activity could be overcome by Vpu [230]; cDNA microarray analyses of messenger RNAs in IFN-α-treated and untreated cells finally identified HM1.24/BST-2/CD317 as the restriction factor, which was re-termed “tetherin” because of its direct tethering function at the cell-surface [231]. Subsequently, it was demonstrated that Vpu-induced down-regulation of tetherin from the cell surface explains its counteraction of the antiviral activity of tetherin [232]. The growing list of enveloped viruses restricted by tetherin includes filoviruses, arenaviruses, paramyxoviruses, gamma-herpesviruses, rhabdoviruses, and a wide array of retroviruses from several mammal host species [233-239]. These restrictions occur not only *in vitro* but also in natural target cells *in vivo*[234,240].

### Antiviral mechanisms of tetherin

Tetherin efficiently blocks the release of Vpu-defective HIV-1 virions by directly tethering them to the surface membranes of virus producer cells. Captured virions are internalized by endocytosis, and subsequently accumulate into CD63-positive endosomes, and probably are degraded in the lysosomes [230,231]. Structurally, the protein’s two membrane anchors formed by the N-terminal TM domain and the C-terminal GPI anchor, together with the conformational flexibility provided by the homodimerized EC domain, are key for the direct tethering mechanism required for the above process. In fact, a totally artificial tetherin-like protein consisting of structurally similar domains from three unrelated heterologous proteins (the CT/TM, EC, and GPI anchor from different proteins) reproduced tetherin's antiviral activity by inhibiting the release of Vpu-deleted HIV-1 and Ebola virus-like-particles, despite their lack of sequence homology with native tetherin [241], suggesting that the configuration of tetherin at the cell surface, but not its primary sequence, is important for the antiviral activity. Lipid raft localization of tetherin, which is determined by the GPI anchor, is in accordance with the preferential site for budding of enveloped viruses [242,243]. Indeed, tetherin has been reported to be enriched at the virological synapse [244], but its role in cell-to-cell transfer of viruses remains controversial [245-249]. As another function, it has very recently been shown that tetherin acts as a viral sensor for the presence of viral infection, inducing NFκB-dependent proinflammatory gene expression [250], an activity that described for TRIM5α [176].

With regard to the configuration of tetherin, several models have been proposed: (1) The EC self-interaction model (Figure 5C) --- individual monomers of tetherin are anchored at both ends (TM and GPI anchor) to the same cellular or viral membrane, and the EC domains of cell-associated and virion-associated monomers are bound through disulfide bonds; (2) The membrane-spanning model (Figure 5D and E), both ends are anchored into the opposite side of the membranes (cellular and viral); and theoretically, the dimerized monomers of tetherin in this model can be formed in either an anti-parallel or parallel configuration. The EC self-interaction and anti-parallel membrane-spanning models are supported by the experiment in which the cleavage of the GPI anchor by enzymatic treatment with Pi–PLC did not relieve the restricted virions at the cell surface, suggesting that each GPI-anchorless monomer that is dimerized still anchors at the different sides of the plasma membrane trough the TM domain [251]. On the other hand, electron microscopy studies have revealed that the actual distance of the gap between viral and cellular membranes is larger than the estimated size of that in the EC self-interaction model [241,252,253]. This evidence strongly supports the (anti-parallel or parallel) membrane-spanning model. Most importantly, combined analyses of high-resolution crystallography and small-angle X-ray scattering-based modeling finally demonstrated that the structure of tetherin’s coiled coil EC domain is indeed a parallel homodimer [252,254-256]. Taken altogether, it is likely that the parallel membrane-spanning configuration model may correspond to the configuration of the antiviral state of tetherin at the cell surface (Figure 5E).

### Action of HIV-1 Vpu

Vpu, which is encoded in the genomes of HIV-1 and a few SIV strains, is an 81-amino acid type I transmembrane protein. It comprises an amino-terminal single TM α-helix domain that also acts as an uncleaved signal peptide, and a carboxy-terminal CT domain in which two cytosolic α-helices are separated by a short flexible connector loop. Vpu mediates proteasomal degradation of CD4 by interacting with newly synthesized CD4 molecules in the endoplasmic reticulum, together with the β-transducin repeat-containing protein (βTrCP) 1 and 2 subunits through its phosphoserine residues in the CT domain [257,258] (Figure 5A and 5F). This βTrCP dependency of Vpu is only partially common to the anti-tetherin activity since βTrCP-binding-defective mutant viruses still retain half of the wild-type activity [259]. Thus, it seems likely that some unknown cellular co-factors other than βTrCP proteins might be required for Vpu to inhibit the antiviral activity of the restriction factor [232,260].

The models of intracellular sites of Vpu’s action in tetherin down-regulation have been controversial. First, it was proposed that Vpu interferes with the membrane transport of newly synthesized tetherin by sequestering the restriction factor in the trans-Golgi network (TGN) [261-263]. Second, Vpu might be able to block the recycling of tetherin by sequestering the latter protein in the recycling endosomes after its internalization from the cell surface [261,263,264]. Third, it was suggested that Vpu might directly internalize tetherin from the cell surface leading to lysosomes [259,265,266], possibly in a cell-type-dependent manner [267]. These three models may not be mutually exclusive, but rather it is likely that each antagonistic model of Vpu is operative to counteract tetherin to varying degrees in different cellular contexts.

In terms of the intracellular fate of tetherin, Vpu-induced down-regulation of the restriction factor might be mediated in part through proteasomal degradation [268-270]. This possibility is based on experiments in which the treatment by proteasomal inhibitors resulted in increased levels of tetherin and loss of Vpu-mediated viral release enhancement. However, prolonged incubation with the inhibitors leads to the depletion of the free ubiquitin pool, affecting both proteasomal and lysosomal degradation [271]. Indeed, the latter degradation pathway has been suggested by evidence that the treatment with inhibitors of the lysosomal pathway blocks the Vpu-mediated tetherin degradation [259,264,266], resulting in a clear colocalization of these two proteins to lysosomal compartments [259,265,267]. In accordance with this, it has been reported that tetherin is constitutively degraded in lysosomes by HRS, a key component of the ESCRT-0 complex that sorts ubiquitinated membrane proteins to lysosomes, and this is accelerated by interaction with Vpu [266]. As another explanation, without inducing any degradation, Vpu simply might sequester either *de novo* or recycled tetherin in the TGN and/or the recycling endosomes plus the TGN, respectively [262,263,272,273], as described above.

The ability of Vpu to bind tetherin through TM-TM interaction is crucial for viral antagonism of this restriction factor [259,268,274,275]. This interaction is highly specific at the amino acid level requiring residues I34, L37, and L41 of tetherin [276] and A14, A18 and W22 of Vpu [277] (Figure 5A and 5F) on the hydrophobic faces of the helices that contribute an interactive surfaces. Recent NMR spectroscopy analysis showed that V30 of tetherin and A10 of Vpu (Figure 5A and 5F) together with the aforementioned residues contribute to form an anti-parallel, lipid-embedded helix-helix interface [278]. Importantly, species specificity of tetherin antagonism by primate Vpu proteins is determined by their TM-TM interaction. Indeed, non-human primate tetherin proteins are mostly insensitive to Vpu antagonism [269,279-281], due to the difference of the amino acid positions 30–45 of the TM sequence [268,279,282] that correspond to the interaction surface as described above.

### Action of SIV Nef

Non-human primate lentiviruses, which lack Vpu, use Nef protein to counteract tetherin’s antiviral function [279,280,283]. The primate ancestors of HIV-1, SIVcpz and SIVgor from chimpanzees and gorillas, which encode Vpu, also use Nef to antagonize their tetherin [283,284]. Interestingly, a very recent report demonstrated that even chimpanzee-adapted HIV-1 molecular clones regained Nef-mediated anti-tetherin activity [285]. While HIV-1 Vpu antagonizes human and chimpanzee, but not other primate tetherin proteins [269,274], SIV Nef counteracts primate but not human tetherin [279,280,283]. This specificity is determined by the CT of non-human primate tetherin, which contains an insertion of five amino acids at positions 14–18 (e.g. DDIWK in chimpanzee) that is responsive to SIV Nef, but is missing in the human counterpart [283,284,286]. Antagonism of non-human primate tetherin is abrogated by SIV Nef mutations that lack the ability to downregulate CD4, implying that its anti-tetherin activity might share some mechanistic properties with CD4 down-regulation [279]. Interestingly, both Vpu and Nef proteins from nonpandemic HIV-1 group O and P viruses lack the activity against human tetherin, while the Nef proteins from these viruses retain the activity against primate tetherins [287].

### SAMHD1

The *SAMHD1* gene was first identified in mice by Lafuse *et al*. They treated mouse peritoneal macrophages by IFN-γ and isolated two genes (*Mg11, Mg21*) from a cDNA library enriched for IFN-γ induced genes. The *Mg21* gene, which encodes an IFN-γ induced GTPase (TGTP), was reported [288]; the *Mg11* gene, which encodes SAMHD1, was directly deposited into Genebank (accession number U15635) (Lafuse, personal communication). Later, the human homologue of *Mg11* was identified from monocyte-derived dendritic cells and was named dendritic cell-derived IFN-γ induced protein (DCIP) [289]. The presence of a sterile alpha motif (SAM) and a histidine-aspartic (HD) domain in DCIP was first noticed when it was found that the expression of this protein was up-regulated in human lung fibroblasts by tumor necrosis factor (TNF)-α [290]. The function of SAMHD1 was not clear, until it was found that this gene mutation caused Aicardi-Goutieres syndrome (AGS) [291]. AGS is an autoimmune disease, which is characterized by elevated type-I interferon production and causes early-onset encephalopathy. These earlier observations suggested that SAMHD1 could play a role in innate immune response to viral infection.

**Figure 6:**
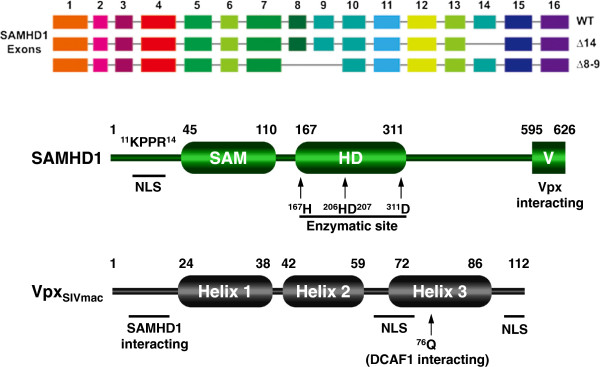
**Schematic illustration of Vpx protein from SIVmac and human SAMHD1 protein.** SAMHD1 splicing variants are shown on the top. Numbers indicate amino acid positions. The three **α-**helices of Vpx and the SAM, HD, and the C-terminal variable region of SAMHD1 are indicated. Other critical residues and motifs include nuclear localization signal (NLS), a critical residue that determines Vpx interaction with DCAF1 (Q76), four critical residues in SAMHD1 NLS (^11^KPPR^14^), and four residues in the HD domain (H167, H206, D207, D311) are all indicated.

Human SAMHD1 has 626 amino acids, which are translated from 16 exons (Figure 6). Two other splicing variants are also produced, which lack exons 8–9 or 14, respectively; but they are much less stable [292]. SAMHD1 comprises an N-terminal nuclear localization domain, which has a nuclear localization sequence (NLS) ^11^KRPR^14^[293,294], a SAM domain (residues 45–110), a HD domain (residues 167–311), and a C-terminal variable domain (Figure 6). The SAM domain is one of the most common protein-protein interaction module of ~70 amino acids, which is found in a variety of signaling molecules including Tyr and Ser/Thr kinases, lipid kinases, scaffolding proteins, RNA binding proteins, transcription factors, and GTPases [295]. The HD domain is found in a variety of enzymes including nucleotidyltransferase, helicase, and dGTPase, indicating that it plays a role in nucleic acid metabolism [296]. SAMHD1 oligomerizes through the HD domain [297], and it binds to nucleic acids through the HD domain [297,298]. SAMHD1 does not have any nuclease activity, but it is a dimeric dGTPase triphosphohydrolase that selectively hydrolyses deoxynucleoside triphosphates (dNTPs), but not ribonucleoside triphosphates (rNTPs) [299,300]. In addition, SAMHD1 is a nuclear protein with its localization determined by the N-terminal NLS. Although SAMHD1 was discovered from dendritic cells, its expression is not limited to myeloid cells, and it is also expressed in lymphoid cells including T and B cells [289,301,302].

### Discovery of SAMHD1 antiretroviral activity

The viral accessory gene *vpr* is only encoded in HIV-1, HIV-2, and SIV, while some SIV strains and HIV-2 additionally express *vpx*, which is duplicated from *vpr*[303,304]. HIV-1 Vpr induces G2 arrest and enhances viral replication in monocyte-derived macrophages (MDMs) [305,306]. Vpx only enhances viral replication in MDMs and monocyte-derived dendritic cells (MDDCs) [307]. Notably, it enhances HIV-1 and MLV replication *in trans* in non-dividing myeloid cells [308,309]. Although both Vpr and Vpx enhance viral replication in MDMs, different mechanisms are involved. While Vpr only enhances viral replication by 2- to 5-fold [310], the activity of Vpx reaches about 100-fold [309,311,312]. In fact, Vpx promotes viral replication at the step of reverse transcription by counteracting a dominant inhibitor [308,309].

Vpr binds to the DDB1-Cul4A-associated-factor-1 (DCAF1) protein, which is a substrate of the Cul4A E3 ligase consisting of Cul4A, RING H2 finger protein homolog (RBX1), and DNA damage-binding protein 1 (DDB1) [313]. This interaction allows Vpr to activate the host DNA-damage-response (DDR) pathway through ATR and initiate G2 arrest. Vpx also interacts with DCAF1, but this interaction is required for Vpx promotion of viral replication [309,312]. It was hypothesized that Vpx triggers proteasomal degradation of an unknown restriction factor via the Cul4A E3 ligase and rescues viral replication at the reverse transcription step in myeloid cells and/or that Vpx promotes viral escape from a proteasomal pathway that is detrimental to viral replication in monocytes-derived dendritic cells [314]. Using an affinity purification procedure followed by mass spectrometry, this unknown restriction factor was identified as SAMHD1 [315-317].

### Action of SAMHD1: depletion of intracellular dNTP pool

The cellular dNTPs can be synthesized either from rNTPs after reduction by the ribonucleoside diphosphate reductase (RNR), or from deoxynucleosides salvaged from degraded DNA after phosphorylation by deoxynucleoside kinases. Because dNTPs are mainly consumed for DNA synthesis, their biosynthesis is S-phase dependent [318]. Thus, non-dividing cells such as MDMs, MDDCs, and resting CD4^+^ T cells have lower intracellular dNTPs, and their levels are greatly elevated in dividing cells such as activated CD4^+^ T cells. Indeed, activated human primary CD4^+^ T cells contain 130 to 250-fold more dNTPs than MDMs [319]. However, HIV-1 is still able to establish a low-level infection in macrophages, because HIV-1 reverse transcriptase (RTase) has ~100-fold higher affinity for dNTPs than MLV RTase, and this allows HIV-1 to synthesize viral DNA even at low dNTP concentrations [320]. Nevertheless, HIV-1 replication is still dependent on intracellular dNTP levels. It was demonstrated a long time ago that increasing dNTP levels in resting peripheral blood lymphocytes (PBLs) could significantly enhance HIV-1 replication [321], and the depletion of cellular dNTP pool by RNR inhibitor hydroxyurea could block HIV-1 replication [322]. SAMHD1 restricts HIV-1 replication in dendritic cells, monocytes, macrophages, and resting CD4^+^ T cells by decreasing the intracellular dNTP levels, resulting in an early post-entry restriction at the level of reverse transcription [301,302,323,324].

The SAMHD1 antiviral activity has been demonstrated by different approaches [325]. The early experiments showed that delivery of Vpx by virus-like particles (VLPs) or directly by virions overcame the HIV-1 restriction in these myeloid cells [309,312,326]. The Vpx proteins were able to specifically trigger SAMHD1 degradation by the proteasomal pathway in these cells [315-317], and a similar observation was also made in resting CD4^+^ T cells [301,302]. The Vpx mutants T17A that did not rescue viral infection and the Q76A mutant that did not bind to DCAF1 were all unable to destabilize SAMHD1. Specific silencing SAMHD1 expression in non-dividing cells by short-hairpin RNAs (shRNAs) increased HIV-1 efficiency; ectopic expression of SAMHD1 in U937-derived macrophages, which is a myeloid cell line and does not express SAMHD1, strongly blocked HIV-1 replication [317,323]. In addition, the depletion of SAMHD1 in these cells resulted in increased intracellular dNTP levels and viral DNA synthesis [301,323,327]. Moreover, monocytes and resting CD4^+^ T cells from AGS patients that do not express functional SAMHD1 proteins were more susceptible to HIV-1 infection [301,302,315]. Furthermore, the antiviral activity of SAMHD1 is not only limited to HIV-1 and SIV, but also extends to other retroviruses including HIV-2, feline immunodeficiency virus (FIV), bovine immunodeficiency virus (BIV), EIAV, and MLV [297]. Collectively, these results demonstrated that SAMHD1 is a Vpx target that strongly blocks viral replication in non-dividing cells by depleting the intracellular dNTP pool. SAMHD1 is a nuclear protein, but its nuclear localization is not required for its enzymatic activity and/or antiviral activity [293,294]. Because cellular dNTPs are not compartmentalized, SMAHD1 should be able to degrade dNTPs in both the cytoplasm and the nucleus (Figure 1). Although SAMHD1 imposes an important block to HIV-1 infection, disruption of this block cannot restore HIV-1 replication in resting CD4^+^ T cells, indicating that there are additional blocks in these cells [301,302]. In addition, another type I IFN-inducible unknown restriction factor in dendritic cells, which also blocks HIV-1 replication at an early step and is counteracted by Vpx, needs to be identified [328].

An important feature of SAMHD1’s antiviral activity is that it requires cells to stay in a resting or non-dividing state. The SAMHD1 activity is only detectable in the myeloid cell lines THP1 and U937 after they are fully differentiated into macrophages by treatment with phorbol myristate acetate (PMA) [323]; although activated primary CD4^+^ T cells still express SAMHD1, this expression neither reduces intracellular dNTP levels, nor does it inhibit HIV-1 replication [302,323]. In addition, ectopic expression of SAMHD1 in a human T cell line did not show these restrictive activities, either [317,323]. Because dividing cells maintain high levels of dNTPs, SAMHD1 may not sufficiently reduce dNTPs to restrict viral replication. Thus, SAMHD1-mediated dNTP hydrolysis and inhibition of viral reverse transcription stand as a very attractive model for SAMHD1 antiviral mechanism in non-dividing cells. Alternatively, the SAMHD1 antiviral activity may not be completely dependent on the dNTP triphosphohydrolase activity. Because SAMHD1 has nucleic acid binding activity, it may interact with viral reverse transcription complex and inhibit production of full-length viral DNA, and this activity may require other cellular factor that is only expressed in non-dividing cells. Thus, the regulation of SAMHD1 antiviral activity remains an important area of future study.

### Action of Vpx

As introduced earlier, Vpx tightly associates with DCAF1, which is a substrate receptor subunit of the Cul4A E3 ubiquitin ligase complex, and this interaction is linked to Vpx activity to relieve SAMHD1 restriction in non-dividing cells [307]. Like Vpr, the Vpx protein has three central α-helices connected by two flexible loops and unstructured amino and carboxy termini (Figure 6) [329,330]. Like SAMHD1, Vpx is also a nuclear protein, which is determined by a C-terminal proline-repeat and a NLS motif crossing the end of second loop and the beginning of the α-helix 3 region [329]. Vpx binds DCAF1 through the α-helix 3 region where the Q76 residue is located [312], and it binds to SAMHD1 through the N-terminal unstructured region, where the T17 residue is located [331,332]. Vpx recognizes the C-terminal 31 amino acid residues of SAMHD1, loads this protein onto the Cul4A-DCAF1 complex, and triggers SAMHD1 proteasomal degradation [331,333]. Indeed, the SAMHD1 C-terminal tail is highly divergent among vertebrate species; so the neutralization of SAMHD1 by Vpx is highly species-specific. For example, Vpx from SIVmac239 can effectively neutralize human but not mouse and zebrafish SAMHD1 [331]. In addition, this domain is the target for strong positive selection during primate evolution, which contains a cluster of five positively selected sites. Among these, the last M626 residue critically determines human and primate SAMHD1 sensitivity to Vpx [333]. Several other positively selected residues are also found in the N-terminal region, and among these, the G46 and R69 also contribute to this species-specific interaction [334]. Notably, although SAMHD1 still retains antiviral activity when it is relocated to cytoplasm, the cytoplasmic SAMHD1 becomes resistant to Vpx-induced degradation [293,294]. Because Cul4A and DCAF1 are also nuclear proteins, which can induce polyubiquitylation of proteins associated with chromatin [335], it is possible that Vpx loads SAMHD1 onto the Cul4A/DCAF1 E3 ligase complex in the nucleus. However, it is still inconclusive whether SAMHD1 is degraded in the nucleus [294], or it is re-targeted to the cytoplasm for degradation [293,333].

HIV-1 does not have the capability to neutralize SAMHD1, because its Vpr does not degrade SAMHD1 and it does not encode a Vpx protein. However, an evolutionary study has uncovered that the ancestral Vpr gene had the ability to antagonize SAMHD1 before it gave rise to the Vpx gene [334]. Accordingly, Vpr proteins from several SIV strains isolated from different old world monkey species are still able to degrade SAMHD1 [294,334]. SAMHD1 may have exhibited evolutionary pressure to differentiate Vpr and Vpx, so that the two proteins have divergent functions. HIV-1 is originally from SIVcpz, whose Vpr does not have SAMHD1-degrading ability [334]. This and other factors may explain why HIV-1 replicates in macrophages at very low levels, and why it cannot infect efficiently dendritic cells [336]. In fact, by not infecting dendritic cells, HIV-1 could avoid activating a cryptic sensor, which induces type I IFNs and thus activates an antiviral response [337]. By evading detection by this sensor, HIV-1 is able to replicate in macrophages at a low level that is sufficient to transmit the virus to activated CD4^+^ T cells. This covert replication strategy may help HIV-1 to establish a persistent infection in humans. In addition, although HIV-1 Vpr does not overcome SAMHD1, it may target another unknown restriction factor in human CD4^+^ T cells, and this mechanism needs to be clarified [338-340].

### MOV10

**Figure 7:**
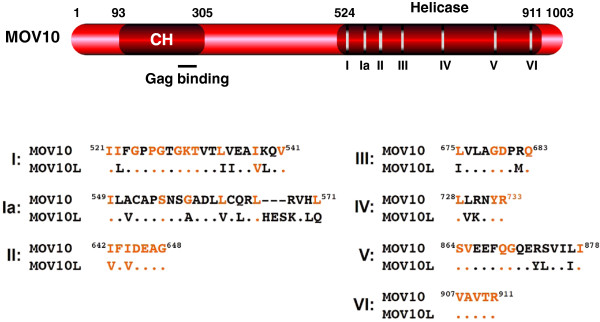
**Schematic illustration of human MOV10 protein.** Numbers indicate amino acid positions. The Cys-His-rich (CH) domain, helicase domain, and seven helicase motifs (I, Ia, II, III, IV, V, VI) are indicated. The amino acid sequences of these motifs from MOV10 and MOV10L proteins are aligned. Dots indicate identical residues, and critical residues in each motif are in orange color.

The Moloney Leukemia Virus 10 inactivated gene *MOV10* was first discovered from the Moloney murine leukemia virus (M-MLV)-carrying mouse strains (Mov mice), which have a single copy of M-MLV provirus at different loci after germline infection [341]. These MOV mice show three different levels of viral replication during development: viremic, conditional viremic, and non-viremic. The MOV10 mouse is non-viremic, because the provirus has mutations in the *gag-pol* region and does not produce infectious particles [342]. The provirus is integrated into a gene locus on chromosome 3, which encodes a 110-kDa protein. Since this protein contains three consensus elements for GTP-binding proteins, it was named gb110 [343]. Later, it was found that this protein has seven conserved helicase motifs, which classified it as a SF-1 helicase [344]. Helicases have purine nucleoside triphosphate phosphatase (ATPase or GTPase) activity, which catalyzes the separation of DNA and/or RNA duplex into single strands in an ATP-dependent reaction [345]. They may have up to seven helicase motifs (I, Ia, II, III, IV, V, and VI) and are classified into three super families (SF-1, SF-2, SF-3) and two small families (F-4, F-5) [346]. Motif I has a GxxxxGKT/S consensus and binds to phosphates; and motif II has a DExx consensus and binds to magnesium (Figure 7). These two motifs catalyze the hydrolysis of purine nucleoside triphosphate, providing energy for helicase activity. The other five motifs are more diverse, and they could contribute to RNA or DNA binding [346]. All helicases have motifs I and II, but only SF-1 and SF-2 helicases have all seven motifs [347]. MOV10 has all seven motifs, and its motif II has a DEAG fingerprint, which qualifies it as a SF-1 helicase [344]. The physiological function of MOV10 was not clear until its ortholog in *Arabidopsis*, the silencing defective gene 3 (SDE3), was found to be required for the RNA silencing pathway [348]. This activity was confirmed by another ortholog in *Drosophila*, the *Armitage* (*Armi*) gene, which is also required for the RNA silencing pathway [349,350]. In addition, MOV10 interacts with the RNA interference machinery through the Argonaute 2 (Ago2) protein in mammalian cells, which further highlights its important role in the regulation of gene expression [351,352].

### Discovery of MOV10 antiretroviral activity

Because the RNA interference (RNAi) pathway defends viral infection in plants, invertebrate, and vetebrate animals [353-355], several components of the mammalian RNAi machinery have been tested for anti-HIV activity [356-360]. Among these proteins, MOV10 was consistently found to have very potent and direct anti-HIV-1 activity when it was ectopically expressed [356,358,360,361]. MOV10 additionally inhibits SIV [360], MLV [360], EIAV [358], hepatitis C virus (HCV) [362], and vesicular stomatitis virus (VSV) [363]. Thus, MOV10 has very broad antiretroviral activity, and this activity may extend to several RNA viruses.

MOV10 has a mammalian paralog, which is called MOV10-like-1 (MOV10L1). MOV10L1 shares 45% amino acid identity with MOV10 in the C-terminal helicase region (Figure 7), and it is specifically expressed in the mouse germ cells [364]. Knockout studies demonstrate that MOV10L1 is required for spermatogenesis by serving as one critical component of the Piwi-interacting RNA (piRNA) pathway, which specifically inhibits retrotransposon activity [365,366]. ~41% of the human genome is constituted from retrotransposons [367], including endogenous retroviruses (ERVs), long-interspersed-element 1 (LINE1), short-interspersed-elements (SINEs)/Alu, and SINE-VNTR-Alu (SVA) [368]. Like retroviruses, the ERVs have two long terminal repeats (LTRs), so they are also called LTR-retrotransposons, and the others are called non-LTR retrotranspnosons (LINE1, SINE-Alu, SINE-SVA). The LTR and LINE1 retrotransposons are strongly activated in the primary spermatocytes of MOV10L1 knockout mice, followed by death of these cells, indicating that MOV10L1 plays a critical role in genome integrity in germ cells. Indeed, MOV10 exhibits similar anti-retrotransposon activity *in vitro*, which inhibits both LTR and non-LTR retrotransposons [361,369,370]. Thus, like A3 proteins, the MOV10 antiviral activity also applies to endogenous retroviral elements. Notably, although both exogenously and endogenously expressed MOV10 proteins inhibit retrotransposon replication [361,369], the endogenous MOV10 was found unable to inhibit HIV-1 replication [361]. This puzzle needs to be solved.

### Action of MOV10

The human MOV10 has 1,003 amino acids, which are translated from 20 or 21 exons in chromosome 1 (Figure 7). Its seven helicase motifs are located in the C-terminal region from residues 524–911. Notably, its N-terminal region from residues 93–305 contains a structurally exposed Cys-His-rich (CH) domain [371], which has been recently recognized as a novel class of protein-protein interaction module [372]. MOV10 decreases both the quantity and quality of the HIV-1 infectious particles in viral producer cells. MOV10 reduces HIV-1 production, possibly by decreasing Gag expression and processing, but this mechanism is still unclear [356]. In addition, MOV10 is packaged into virions and inhibits HIV-1 replication from the 2^nd^ cycle by interfering with viral reverse transcription (Figure 1) [356,358,360]. The MOV10 packaging involves a specific interaction with Gag. MOV10 interacts with Gag in the NC region, probably via the basic linker domain [371]. On the other hand, Gag binds to the MOV10 CH domain via a region from amino acid 261–305 [371]. However, the CH-domain is not sufficient for packaging of the full-length MOV10 protein, and its packaging also requires the C-terminal helicase motifs [371]. Because these helicase motifs have high-affinity for RNA, unknown cellular RNAs are required for MOV10 packaging. In fact, MOV10 is packaged inside the core [360], which allows MOV10 to directly interact with viral RNA and block viral reverse transcription in the target cells. Compared to its reduction of viral production, the reduction of viral infectivity by MOV10 is more significant, leading to over 100-fold inhibition of viral replication. MOV10 also associates with retrotransposon ribonucleoprotein particles (RNPs) and inhibits their replication in a similar manner [369,370]. LINE1 produces two proteins: a 40-kDa RNA-binding protein ORF1p, and a 150-kDa ORF2p protein that has endonuclease and reverse transcriptase activities. MOV10 tightly interacts with ORF1p, which mediates its strong anti-LINE1 activity [369]. Because all these inhibitory activities require its helicase domain, MOV10 likely recognizes a common RNA secondary structure to exhibit its inhibitory effect. Unlike other restriction factors, MOV10 has not been subjected to positive selection, indicating that it may not participate into the co-evolutionary arms race with exogenous pathogens. However, its strong sequence conservation across species suggests that MOV10 may play an important role *in vivo*[369].

### MicroRNAs

While the primary aim of this review is to survey protein restriction factors, one should be mindful that non-coding RNAs and RNAi activities have also been found to play increasingly significant regulatory and effector roles in eukaryotic biology. Indeed, RNAi activity is ubiquitously involved in normal and diseased physiology including cancers, metabolic disorders, and infectious diseases [373-375]. In the realm of host-virus interaction, it was originally thought that RNAi only serves host defense against viral infection in plants and invetebrate animals [376,377]; however, emerging evidence suggests that this defense also functions in mammals [353,378]. Significant findings supportive of this notion arise from evidence that the virulence of viral infection in mammals is exacerbated by a reduction in host RNAi function [348,355,379-381].

MicroRNAs (miRNAs) represent a major class of small non-coding RNAs in the human genome. Humans encode for more than 1,600 characterized miRNAs (miRbase.org). Relevant to HIV-1, many human miRNAs have been found to directly target HIV-1 sequences and to attenuate virus replication in cells. These include miR-28, miR-29a, miR, miR-125b, miR-150, miR-223, miR-382, miR-133b, miR-138, miR-149, and miR-326 [382-388]. Other cellular miRNAs have also been shown to indirectly target factors such as PCAF and cyclin T1 that are needed by HIV-1 to replicate [355,389,390]. In this manner, these miRNAs can indirectly repress HIV-1 replication in cells [391]. MiRNA-repression of the intracellular replication of mammalian viruses appears to be a common theme; indeed, an increasingly large number of published reports document the suppression by various human miRNAs of Epstein Barr Virus (EBV) [392,393], Kaposi’s sarcoma Herpes virus (KSHV) [394], hepatitis B virus [395,396], coxsackie virus [397], human papilloma virus [398], amongst others. This list of examples promises to grow longer over time.

In view of the above, how do viruses counter the host cell’s RNAi restriction? In principle, there are several means that viruses can employ, including the shielding of viral genomes from access by RNAi, the mutation of viral sequences to evade RNAi, the encoding of viral RNAi suppressor moieties, and changing the miRNA expression profile of the infected cells [399]. For HIV-1, several published reports have shown that the virus can explicitly alter the cellular profile of miRNA expression [400,401], presumably to benefit viral replication. Other reports have implicated that the HIV-1 Tat protein [402-407] and the viral TAR RNA [408] serve RNAi-suppressing activities. Tat, like the HTLV-1 Rex protein, likely suppresses RNAi through sequestration of RNA via its basic amino acids [409]. Nevertheless, the RNAi-suppressing activity of Tat appears to be modest and has been difficult to measure in some assays [410].

Several HIV-1 encoded small non-coding RNAs (ncRNAs) have also been identified in infected cells using next generation pyrosequencing; and the over expression of these ncRNAs represses viral replication [411-413]. HIV-1, like HTLV-1 [414,415], also expresses antisense non-coding RNAs [416,417]. Currently, we do not fully understand the roles of these non-coding HIV-1 RNAs. The clarification of their biological functions in virus replication represents an important future challenge for investigators.

### Conclusions

Over the past decade much progress has been made in generating insights into HIV-1 virus-host interactions. In this respect, several hundred host dependency factors have been identified that act positively to regulate HIV-1 replication in human cells [418-423]. As a counterweight to the study of positive host factors, it is also instructive and important to appreciate the role that restriction factors play in moderating HIV-1 replication. Our survey here of several examples of HIV-1 restriction factors is not intended to be complete or fully comprehensive. We hope the review provides a platform that introduces this topic to those readers interested in further studies of viral restriction factors.

## Competing interests

The authors declare that they have no competing interests.

## Authors’ contributions

YHZ, KTJ, and KT wrote different sections of this manuscript. YHZ and KT prepared the figures. All authors read and approved the final manuscript.
